# Elevated levels of interleukin-12/23p40 may serve as a potential indicator of dysfunctional heart rate variability in type 2 diabetes

**DOI:** 10.1186/s12933-021-01437-w

**Published:** 2022-01-06

**Authors:** A. M. Wegeberg, T. Okdahl, S. Riahi, N. Ejskjaer, F. Pociot, J. Størling, B. Brock, C. Brock

**Affiliations:** 1 Mech-Sense, Department of Gastroenterology and Hepatology, Aalborg University Hospital and Clinical Institute, Aalborg University, Aalborg, Denmark; 2 Thisted Research Unit, Aalborg University Hospital Thisted, Thisted, Denmark; 3 Department of Cardiology, Aalborg University Hospital, Aalborg, Denmark; 4 Steno Diabetes Center North Denmark, Aalborg University Hospital, Aalborg, Denmark; 5 Department of Endocrinology, Aalborg University Hospital, Aalborg, Denmark; 6 Steno Diabetes Center Copenhagen, Gentofte, Denmark

**Keywords:** Diabetes mellitus, type 2, Cardiovascular diseases, Inflammation

## Abstract

**Background:**

Systemic inflammatory processes plausibly contribute to the development of cardiovascular complications, causing increased morbidity and mortality in type 2 diabetes. Circulating inflammatory markers, i.e., interleukin (IL)-6 and tumour necrosis factor-α, are associated with neurocardiac measures. We examined a broad panel of various inflammatory and inflammation-related serum markers to obtain more detailed insight into the possible neuro-immune interaction between cardiovascular regulation and systemic level of inflammation.

**Methods:**

Serum samples from 100 participants with type 2 diabetes were analysed. Heart rate variability, cardiovascular autonomic reflex tests, and cardiac vagal tone tests were performed based on electrocardiographic readings. Data regarding covariates (demographic-, diabetes-, and cardiovascular risk factors) were registered.

**Results:**

Increased serum levels of IL-12/IL-23p40 (p  < 0.01) and intercellular adhesion molecule (ICAM)-1 (p  < 0.007) were associated with diminished heart rate variability measures. After all adjustments, the associations between IL-12/23p40, SDANN and VLF persisted (p  = 0.001). Additionally, serum levels of vascular endothelial growth factor (VEGF)-C were associated with response to standing (p  = 0.005).

**Discussion:**

The few but robust associations between neurocardiac regulation and serum markers found in this study suggest systemic changes in proinflammatory, endothelial, and lymphatic function, which collectively impacts the systemic cardiovascular function. Our results warrant further exploration of IL-12/IL-23p40, ICAM-1, and VEGF-C as possible cardiovascular biomarkers in T2D that may support future decisions regarding treatment strategies for improved patient care.

**Supplementary Information:**

The online version contains supplementary material available at 10.1186/s12933-021-01437-w.

## Introduction

Cardiovascular disease (CVD) is the leading cause of morbidity and mortality in type 2 diabetes (T2D), accounting for approximately 50% of all deaths [1]. Cardiovascular functions, including heart rate, heart contractility, and blood pressure, are regulated by autonomic sympathovagal balance [2]. In diabetes, this balance may be tilted by diabetic neuropathy, which can impact the body's neuronal regulation both peripherally, centrally, and within the autonomic nervous system, leading to debilitating, body encompassing complications [3]. To assess the impact of autonomic cardiac regulation, heart rate variability measured during rest and stressors (cardiovascular autonomic reflex tests) have been applied. Heart rate variability index neurocardiac adaptability based on fluctuations of subsequent heartbeats [2]. As a reduction in neurocardiac function is known to precede symptoms, these tests are currently used as the clinical standard to diagnose cardiovascular autonomic neuropathy [4].

Inflammation is an underlying pathogenic component in T2D, along with hyperglycemia, hyperlipidemia, and hypertension, adding to endothelial dysfunction and accelerated atherosclerosis [5–7]. Systemic inflammation is assessed by serum levels of proinflammatory cytokines such as tumor necrosis factor (TNF)‐α and interleukin (IL) such as IL‐6. In neurodegenerative diseases, e.g., in Alzheimer’s disease [8], such systemic inflammation is known to associate with neuronal loss, resulting in widespread disruption of neuronal function, and over the last decade, accumulating evidence suggests that underlying chronic inflammation also contributes to the development of diabetes complications including neuropathy and CVD [9]. In both in recent-onset and long-term T2D, associations between cardiac measures and inflammatory markers highlight changes in proinflammatory cytokines like interleukin-6 [10–12], TNF-α [13], and IL-18 [10, 14], as well as intercellular adhesion molecule (ICAM)-1 [14, 15], and C-reactive protein (CRP) [10, 14] as potential contenders for investigating modulation of neurocardiac function. The proposed foundation for this neuro-immune interaction is the vagally innervated, cholinergic, anti-inflammatory reflex which modulates and maintains homeostasis within the inflammatory and neurocardiac axes [16, 17]. Hence, dysregulation within this interaction could alter cardiac and inflammatory function, impeding neurocardiac health.

The investigated markers have so far been few. Hence, this study set out to examine a broad panel of various inflammation and inflammation-related serum markers to obtain more detailed insight into the possible neuro-immune interaction between cardiovascular health and the inflammatory state. Our data provide evidence for novel associations between inflammatory serum markers and neurocardiac dysfunction, further supporting that inflammatory mechanisms could be critical drivers of CVD in T2D.

## Methods

### Study population

Adults with T2D were recruited consecutively between March 2018 and August 2020 from the outpatient clinic at the Department of Endocrinology, Aalborg University Hospital, Denmark, through advertisement at the local Diabetes Association or online campaigns. Eligible participants were  ≥ 18 years old, of Northern European descent, with verified T2D for more than 1 year (HbA1c  ≥ 6.5%). Participants were on stable antihyperglycemic medication, including metformin and insulin, according to national guidelines. Exclusion criteria included symptomatic ischemic heart disease or cardiac heart failure, treatment for endocrinological diseases other than diabetes, known psychiatric or neurological disease other than diabetic neuropathy, present or previous chemotherapy or use of drugs that affect the nervous system, known abuse (present or previous) of alcohol or medicine (alcohol intake within recommendation was allowed), and celiac disease. The study was conducted in accordance with the Declaration of Helsinki, local regulations, and the International Conference on Harmonization Good Clinical Practice guidelines. The North Denmark Region Committee on Health Research Ethics, Denmark (N-20170045) approved the protocol, amendments, and informed consent form. All subjects gave their informed consent.

### Serum markers

Fasting blood samples were drawn from the cubital vein of the participants in serum tubes before cardiac measures were performed. Samples were centrifuged at 2500 rpm for 10 min, and serum was stored in a − 80 °C freezer until analysis. Serum concentrations of angiogenetic markers [basic fibroblast growth factor (bFGF), fms related receptor tyrosine kinase(Flt)-1, phosphatidylinositol-glycan biosynthesis class F protein (PlGF), tyrosine-protein kinase(Tie)-2, vascular endothelial growth factor (VEGF)-C, VEGF-D], cytokines (IL-5, IL-7, IL-12/IL-23p40, IL-15, IL-16, IL-17A, TNF-β, VEGF-A), chemokines [Eotaxin, Eotaxin-3, interferon gamma-induced protein(IP)-10, macrophage-derived chemokine(MCP)-1, MCP-4, monocyte chemoattractant protein (MDC), macrophage inflammatory protein (MIP)-1α, MIP-1β, thymus and activation regulated chemokine (TARC)], proinflammatory cytokines [interferon(IFN)-γ, IL-2, IL-4, IL-6, IL-8, IL-10, TNF-α], and vascular injury markers [CRP, ICAM-1, soluble vascular adhesion molecule(VCAM)-1, serum amyloid A (SAA)] were analysed using V-PLEX Neuroinflammation panel (Meso Scale Diagnostics, Rockville, Maryland, USA) in duplicates according to the manufacturer’s protocol. Readings with a value of zero or a coefficient of variation between duplicates above 30% were excluded from the dataset. Samples below detection level were transformed by dividing the detection level value by the square root of 2 [18]. If a sample exceeded three standard deviations from the group mean, it was considered an outlier and removed before analysis.

### Cardiac measurements

#### Heart rate variability

Multiple day electrocardiograms were recorded using an extended Holter monitor, ePatch (BioTelemetry Technologies ApS, Hørsholm, Denmark), mounted on the sternum of the participant. The electrocardiograms were imported into Cardiscope™ ANALYTICS (Professional Edition, HASIBA medical GmbH, Graz, Austria) and automatically edited for artifacts. Only recordings with a minimum length of 24 h and a percentage of valid sinus rhythm (a measure of data quality) higher than 90% (excluding sinus tachycardia, bradycardia, and artifacts) were used in the statistical analysis [19]. The following parameters were extracted from the 24-h heart rate variability analysis and interpreted according to international guidelines [4]: time-derived measures [SDNN, the standard deviation of the averages of inter-beat interval (SDANN), mean standard deviation of the averages of inter-beat interval for each 5-min interval (SDNNi), root mean square of the successive differences (RMSSD)] and frequency domain measures [very low frequency (VLF), low frequency (LF), high frequency (HF)].

#### Cardiovascular autonomic reflex test

Electrocardiographic recordings during rest and stressor comprising expiration:inspiration ratio (deep breathing), Valsalva ratio, and 30:15 supine to standing ratio (postural change) was performed using the Vagus™ (Medicus Engineering ApS, Aarhus, Denmark), and has been described in detail elsewhere [20]. The cardiovascular autonomic neuropathy score was defined as established CAN when two or more tests were abnormal, borderline CAN when one test was abnormal, and no CAN when the obtained tests were within the normal range of the specific age-dependent cut-off values [21].

#### Cardiac vagal tone

A 5-min, standard 3-lead electrocardiographic recording [eMotion Faros 180 device (Bittium, Oulu, Finland)] was used to compute cardiac vagal tone using the validated algorithm from the ProBioMetrics online app (version 1.0, ProBioMetrics, Kent, United Kingdom) [22]. Recording artifacts were defined as a sudden change in two succeeding heartbeats exceeding 15 beats per minute in variation (e.g., coughing or movement). In this case, files were cleaned by removing five heartbeats before and after to derive the true cardiac vagal tone [20].

### Clinical measurements

Cohort characteristics comprising age, sex, disease duration, smoking habits, diabetes, and medications were obtained directly from the participants. Height was measured using a stadiometer (Seca GmbH & Co. KG., Hamburg, Deutschland). Weight was assessed with a standardised calibrated scale (BWB-800AS, Tanita, Arlington Heights, Illinois, USA). Body mass index was calculated from these. Heart rate, resting systolic, and diastolic blood pressures were obtained in a seated position using a blood pressure monitor (Intellisense^®^, Omron Healthcare, Inc., Bannockburn, Illinois, USA). Mean arterial blood pressure (MAP) was calculated as (2 × systolic  +  diastolic)/3. Fasting blood samples for biochemical assessment of blood glucose, haemoglobin A1c levels, and lipid profiles were drawn according to standard laboratory procedures and analysed at the Department of Clinical Biochemistry, Aalborg University Hospital.

### Statistics

Data are presented as mean  ±  standard deviation, median (25–75th percentiles), or number (%), dependent on data type and distribution. Data was analysed using an “intention-to-treat” approach. Linear regression was performed with neurocardiac measures (heart rate variability, cardiac vagal tone, cardiovascular autonomic neuropathy, and cardiovascular autonomic reflex tests ratios) as dependent and serum markers (angiogenesis, cytokines, chemokines, proinflammatory, and vascular injury panels) as independent variables. Significant associations were adjusted for covariates at three levels: (1) demographic factors—age and sex; (2) diabetes factors—disease duration, haemoglobin A1c, and use of insulin; (3) cardiovascular risk factors—body mass index, smoking (current, party, and previous as one), hypertension (systolic above 140 or diastolic above 40) and use of antihypertensive medications, as well as the three levels combined (all). A significance level of p  < 0.01 was set to correct for multiple comparisons. All statistical analyses were performed in Stata (version 15 and 16, StataCorp, College Station, TX, USA).

## Results

### Study population

**Fig. 1:**
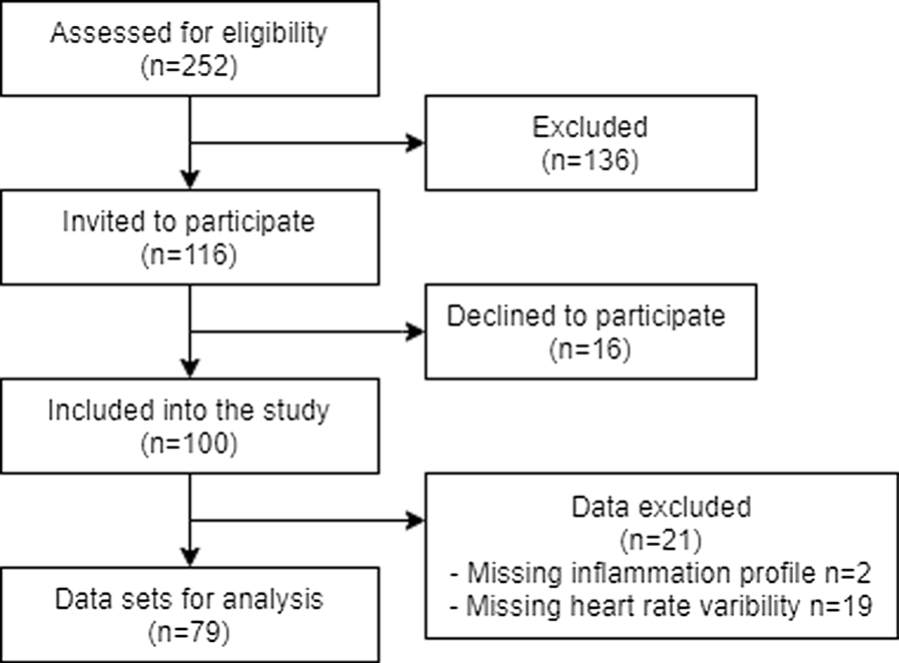
Flowchart of participant selection. Participants were invited to participate between March 2018 and August 2020 through advertisement at the local Diabetes Association, online campaigns or when visiting the outpatient clinic. Two-hundred-and-fifty-two eligible participants showed interest in participation. Of these, 136 did not fulfill the inclusion and exclusion criteria. Additional, 16 declined to participate after receiving further information about the study. In total 100 individuals with diabetes were included into the study

One hundred participants between the ages of 31 and 84 (median: 65 years) diagnosed with T2D for 1–28 years (median: 10 years) before study inclusion were included. A flow chart that detail participant inclusion is depicted in Fig. 1. The use of antihyperglycemic medications were reported by 93%, of which the majority used biguanides, while 26% used insulin (long-acting dose median 50 IU, short-acting dose median 22 IU). Despite this, 75% were hyperglycaemic at the time of investigations (glucose  > 7.8 mmol/L or HbA1c  > 48 mmol/mol). Antihypertensive medications were used by 68%, while statins were used by 70%. Previous smoking was reported by 40%, with only 5% reporting smoking at the time of the study. Cardiovascular autonomic reflex tests revealed 31% to have borderline and 9% to have established cardiovascular autonomic neuropathy, while the median cardiac vagal tone was 3.2 on a linear vagal scale. Participant characteristics are shown in Table 1. For more details and data on heart rate variability measures and serum marker levels, see Additional file 1: Table S1a, b.

**Table 1:** Participant characteristic

Demographic data	
Age (years)	65 (57; 71)
Sex (male)	63%
Body mass index (kg/m^2^)	31.4 ± 5.6
Normal range (18–25)	13%
Overweight (25–30)	27%
Obese class I (30–35)	36%
Obese class II–III (> 35)	24%
Disease duration (years)	10 (5; 17)
Smoking habits	
Current smoking (yes)	3%
Social smoking (yes)	2%
Previous smoking (yes)	40%

Medications	
Antidiabetic medications (yes)	93%
Biguanides	78%
SGLT-2 inhibitors	25%
DPP-4 inhibitors	18%
Sulfonylureas	9%
Insulin (yes)	26%
Long-acting	26%
Dose (IU)	50 (40; 92)
Short-acting	7%
Dose (IU)	22 (9; 100)
Antihypertensives (yes)	68%
ACE-inhibitor	32%
β-blocker	17%
Statins (yes)	70%

Biochemistry	
Fasting glucose levels (mmol/L)	8.9 (7.5; 10.4)
Haemoglobin A1c (mmol/mol)	55 (48; 62)
Cholesterol (mmol/L)	3.9 ± 0.9
Triglyceride (mmol/L)	1.4 (1.0; 2.0)
Low density lipoprotein (mmol/L)	1.9 (1.4; 2.4)
High density lipoprotein (mmol/L)	1.2 (1.0; 1.5)
eGFR	89 (73; 90)
Creatinin	8607 (5774; 13,190)
Albumin	0.01 (0.006; 0.019)
ALAT	27 (21; 37.5)

*SGLT* sodium-glucose linked transporter; *DPP* dipeptidyl peptidases; *ACE* angiotensin-converting enzyme; *eGFR* estimated glomerular filtration rate; *ALAT* alanine aminotransferase

### Serum markers association with neurocardiac function

Out of 34 examined serum markers, we successfully measured six angiogenetic markers (bFGF, Flt-1, PIGF, Tie-2, VEGF-C, VEGF-D), six chemokines (Eotaxin, IP-10, MCP-1, MDC, MIP-1β, TARC), five cytokines (IL-7, IL-12/IL-23p40, IL-15, IL-16, VEGF-A), five proinflammatory markers (IL-6, IL-8, IL-10, IFN-γ, TNF-α), and four markers of vascular injury (CRP, sICAM1, sVCAM1, SAA), in more than 94% of the samples. In contrast, the remaining serum markers were either below the detection threshold, only detected in very few samples, or had a coefficient of variation between duplicates above 30% and were thus not included in the analysis. Complete data on association and adjustments can be found in Tables 2, 3 and Additional file 1: Table S4.

**Table 2:** Associations between neuroinflammatory markers and heart rate variability

	SDNN		SDANN		SDNNi		rMSSD		VLF		LF		HF		LF/HF	
	R^2^	p	R^2^	p	R^2^	p	R^2^	p	R^2^	p	R^2^	p	R^2^	p	R^2^	p
Angiogenesis																
bFGF	0.003	0.60	0.001	0.75	0.046	0.05	0.005	0.51	0.046	0.05	0.025	0.15	0.023	0.16	0.005	0.53
Flt-1	0.003	0.61	0.000	0.97	0.000	0.96	0.004	0.54	0.000	0.90	0.004	0.54	0.008	0.40	0.009	0.39
PIGF	0.003	0.60	0.002	0.66	0.011	0.32	0.002	0.70	0.005	0.50	0.012	0.31	0.006	0.49	0.010	0.34
Tie-2	0.001	0.84	0.019	0.20	0.000	0.98	0.002	0.67	0.000	0.94	0.007	0.44	0.003	0.59	0.011	0.32
VEGF-C	0.011	0.33	0.001	0.82	0.000	0.97	0.000	0.95	0.001	0.79	0.007	0.45	0.001	0.82	0.000	0.87
VEGF-D	0.000	0.97	0.003	0.61	0.001	0.79	0.000	0.92	0.000	0.92	0.005	0.53	0.000	0.87	0.006	0.49
Chemokine																
Eotaxin	0.019	0.21	0.051	0.04	0.038	0.07	0.000	0.96	0.032	0.09	0.024	0.14	0.004	0.73	0.019	0.20
IP-10	0.026	0.14	0.008	0.41	0.032	0.10	0.003	0.60	0.030	0.11	0.032	0.10	0.000	0.91	0.026	0.14
MCP-1	0.001	0.79	0.001	0.82	0.013	0.30	0.004	0.55	0.014	0.27	0.010	0.35	0.021	0.18	0.002	0.72
MDC	0.043	0.05	0.022	0.18	0.046	0.05	0.017	0.23	0.061	0.02	0.047	0.04	0.009	0.38	0.026	0.14
MIP-1β	0.003	0.58	0.002	0.67	0.001	0.80	0.005	0.52	0.000	0.89	0.004	0.58	0.001	0.76	0.004	0.54
TARC	0.019	0.20	0.000	0.89	0.001	0.79	0.013	0.29	0.002	0.66	0.006	0.50	0.000	0.91	0.025	0.14
Cytokine																
IL-7	0.013	0.29	0.030	0.11	0.013	0.30	0.000	0.83	0.008	0.42	0.015	0.26	0.011	0.33	0.013	0.29
IL-12/IL-23p40	**0.074**	**0.010**	**0.101**	**0.003**	**0.081**	**0.007**	0.007	0.45	**0.095**	**0.003**	0.048	0.04	0.015	0.26	0.029	0.11
IL-15	0.022	0.17	0.008	0.43	0.003	0.63	0.000	0.96	0.000	0.99	0.012	0.32	0.001	0.80	0.036	0.08
IL-16	0.003	0.59	0.007	0.44	0.000	0.98	0.004	0.58	0.001	0.82	0.006	0.48	0.001	0.81	0.017	0.23
VEGF-A	0.000	0.99	0.000	0.97	0.008	0.40	0.011	0.34	0.005	0.51	0.009	0.39	0.007	0.44	0.003	0.62
Proinflammatory																
IL-6	0.051	0.04	0.075	0.011	0.033	0.09	0.000	0.091	0.016	0.25	0.019	0.20	0.000	0.89	0.045	0.05
IL-8	0.000	0.85	0.000	0.86	0.008	0.42	0.000	0.97	0.011	0.33	0.012	0.31	0.002	0.69	0.000	0.85
IL-10	0.030	0.10	0.024	0.14	0.039	0.06	0.019	0.20	0.052	0.03	0.015	0.26	0.027	0.13	0.001	0.77
IFN-γ	0.000	0.90	0.000	0.89	0.031	0.11	0.010	0.37	0.029	0.11	0.036	0.08	0.012	0.31	0.005	0.50
TNF-α	0.033	0.10	0.015	0.27	0.019	0.21	0.002	0.66	0.018	0.21	0.002	0.71	0.002	0.72	0.006	0.48
Vascular injury																
CRP	0.026	0.13	0.051	0.04	0.012	0.30	0.000	0.90	0.008	0.41	0.019	0.21	0.000	0.97	0.026	0.14
sICAM1	**0.081**	**0.007**	**0.104**	**0.002**	0.050	0.03	0.000	0.89	0.066	0.015	0.039	0.63	0.003	0.61	0.019	0.20
sVCAM1	0.025	0.14	0.034	0.09	0.034	0.09	0.013	0.29	0.031	0.10	0.008	0.43	0.017	0.23	0.000	0.93
SAA	0.049	0.04	0.064	0.018	0.026	0.14	0.002	0.65	0.015	0.26	0.035	0.08	0.010	0.35	0.006	0.47

A p value ≤ 0.01 is considered significant and marked in bold

**Table 3:** Associations adjusted for demographic factors, diabetes factors and cardiovascular risk factors

	Crude		Demographic		Diabetes		Cardiovascular		All	
	R^2^	p	R^2^	p	R^2^	p	R^2^	p	R^2^	p
SDNN										
IL-12/IL-23p40	**0.074**	**0.010**	0.077	0.013	0.171	0.04	0.147	0.019	0.236	0.07
sICAM-1	**0.081**	**0.007**	**0.087**	**0.008**	0.173	0.03	0.134	0.037	0.227	0.10
SDANN										
IL-12/IL-23p40	**0.101**	**0.003**	**0.103**	**0.004**	**0.156**	**0.010**	**0.126**	**0.006**	0.191	0.03
sICAM-1	**0.104**	**0.002**	**0.111**	**0.003**	**0.161**	**0.007**	**0.120**	**0.007**	0.193	0.03
SDNNi										
IL-12/IL-23p40	**0.081**	**0.007**	0.093	0.013	0.100	0.014	**0.185**	**< 0.001**	**0.247**	**0.001**
VLF										
IL-12/IL-23p40	**0.095**	**0.003**	**0.131**	**0.009**	**0.111**	**0.006**	**0.199**	**< 0.001**	**0.282**	**0.001**
RS										
VEGF-C	**0.083**	**0.005**	**0.208**	**0.001**	**0.154**	**0.003**	**0.150**	**0.003**	**0.291**	**< 0.001**

Data is noted as R-squared and p values for each regression analysis. A p value  ≤ 0.01 is considered significant and marked in bold. Raw: unadjusted analysis. Demographic: adjusted for demographic factors: age and sex. Diabetes: adjusted for diabetes factors: disease duration, haemoglobin A1c and use of insulin. Cardiovascular: adjusted for cardiovascular risk factors: BMI, smoking, hypertension, and use of antihypertensive medications. All: adjusted for both demographic, diabetes and cardiovascular

*SDNN* standard deviation of all normal inter-beat interval; *SDANN* standard deviation of the averages of interbeat intervals; *SDNNi* mean standard deviation of the averages of inter-beat interval for each 5-min interval; *VLF* very low frequency; *IL* interleukin; *sICAM* soluble intercellular adhesion molecule; *VEGF* vascular endothelial growth factor

**Fig. 2:**
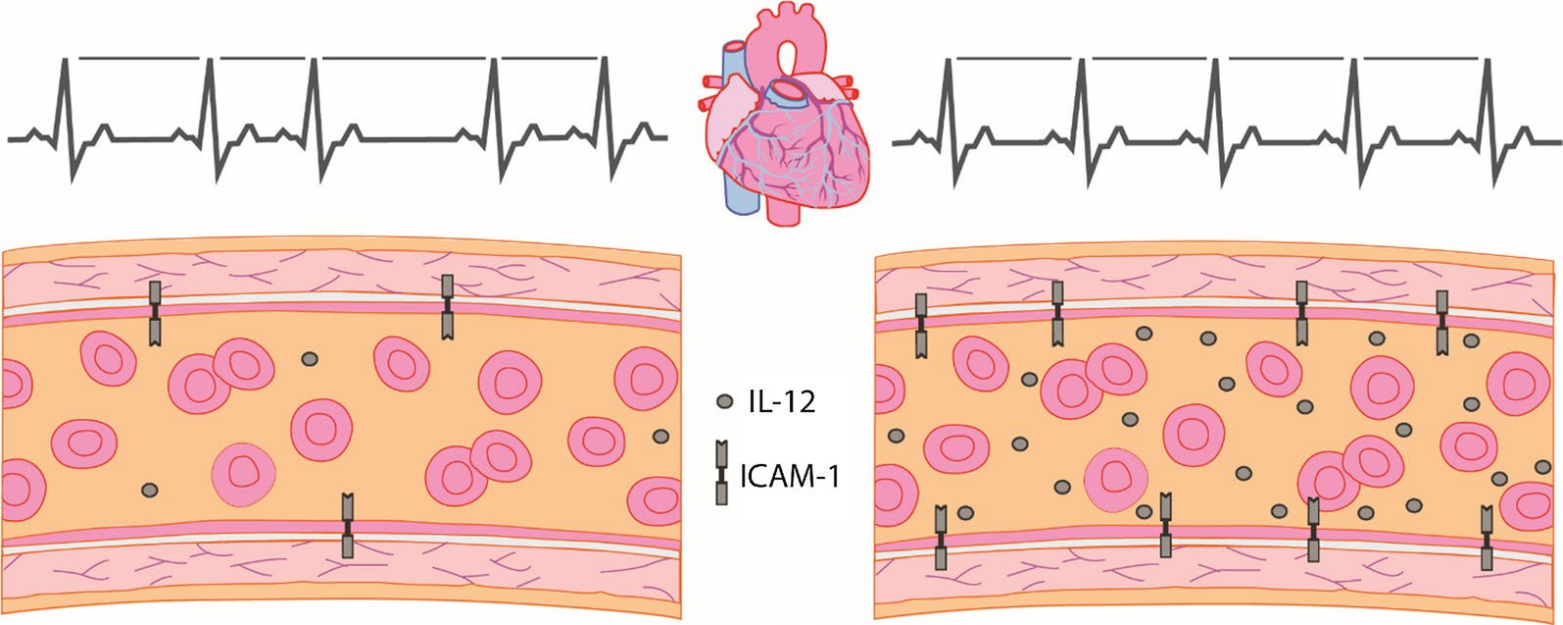
Heart rate variability changes with levels of inflammatory serum markers. Left: normal levels of IL-12 and ICAM-1 associated with normal heart rate variability. Right: increased levels of IL-12 and ICAM-1 resulting in decreased heart rate variability. IL-12 is known to associate with arterial stiffness in healthy [28]

The T-cell differentiating cytokine IL-12/IL-23p40 was inversely associated with time-domains of SDNN (p  = 0.010), SDANN (p  = 0.003), and SDNNi (p  = 0.007), as well as frequency domain of VLF (p  = 0.003), which represents parasympathetic tone [23]. The associations with SDANN and VLF remained significant after individual adjustments for demographic factors, diabetes factors, and cardiovascular risk factors (p  < 0.009), while SDNNi dissipated when adjusting for demographic factors (p  = 0.013) and diabetes factors (p  = 0.013) and SDNN dissipated for all adjustments (p  > 0.013). Only associations with SDNNi and VLF persisted after the combined adjustment (p = 0.001). There was no association between the remainder cytokines or proinflammatory markers and neurocardiac function (Fig. 2).

The endothelial adhesion molecule ICAM-1 was inversely associated with time-domains of SDNN (p  = 0.007) and SDANN (p  = 0.002). The association with SDANN remained significant after individual adjustment for demographic factors, diabetes factors, and cardiovascular risk factors (p  < 0.007), while SDNN dissipated when adjusting for diabetes factors (p  = 0.04) and cardiovascular risk factors (p  = 0.019). None of the associations persisted after the combined adjustment (p  > 0.03). There were no associations between the remaining vascular injury markers and neurocardiac function.

The angiogenesis marker VEGF-C was associated with the resting to standing ratio (p  = 0.005). This association remained after individual adjustment for demographic factors, diabetes factors, and cardiovascular risk factors (p  < 0.003) and combined adjustments (p  < 0.001). There were no associations between the remaining angiogenesis markers and neurocardiac function.

No associations between any of the chemokines and neurocardiac function were found.

## Discussion

In this cohort of adults with T2D, we found that increased levels of IL-12/IL-23p40 and ICAM-1 were associated with diminished cardiac-derived time-domain measures of heart rate variability, suggesting parasympathetic withdrawal on autonomic regulation. Additionally, the level of VEGF-C was indicative of a changed response to standing, suggesting an interplay with the adaptive mechanism of blood pressure changes. These findings were uninfluenced by demographic factors, diabetes factors, and cardiovascular risk factors, as the associations persisted after adjustments.

Diabetes is a prime risk factor for developing CVD [1]. Still, the clinical links between diabetes and CVD have complex and multifactorial pathophysiology, including metabolic stress and neuroinflammatory processes [24]. Indeed, T2D is primarily considered a state of chronic, low-grade inflammation. Evidence suggests that this inflammatory state, aided in most cases by excess adipose tissue, precedes insulin resistance in pre-diabetic subjects, ultimately constituting the factor that increases CVD risk in disease progression [9]. Inflammation is one of the main characteristics of diabetic peripheral neuropathy [25], and it would be reasonable that the same mechanisms are at play in the autonomically innervated cardiovascular system. Hitherto, the focus has mainly been around proinflammatory cytokines such as TNF-α, IL-6, and IL-18, where increased serum marker levels have been shown to associate with autonomic imbalance as detected by heart rate variability or dynamic reflex tests [10, 11, 13, 14]. Interestingly, we found no associations between neurocardiac function and these markers in the current study. This discrepancy could lie in the use of multiplex technology, found to have a good sensitivity at a low detection limit and a broad dynamic range [26] compared to traditional Elisa used in previous studies. Alternatively, it could be found in the differences between the study populations. Our cohort was slightly older and had a longer disease duration than the other studies, some of which investigated recent-onset T2D. Additionally, our cohort had slightly better glycemic control than previously investigated cohorts.

We found a strong association between increased IL-12/IL-23p40 and neurocardiac function. IL-12 and IL-23 belong to the same interleukin family sharing the p40 subunit, enabling proinflammatory effects on Th1 and Th17 [27]. This family is involved in differentiating T cells into Th cells and is known to be elevated in CVD [27]. Accumulated levels of IL-12 have also been found in atherosclerotic plaques and are associated with arterial stiffness and myocardial infarction in healthy and other disease types [28]. However, based on currently available data, we cannot exclude if the same is evident in diabetes. Concurrently, elevated levels of IL-23 are associated with carotid atherosclerosis, lower extremity peripheral arterial disease, and ischemic heart attack [27], but this is the first time it has been reported in T2D patients. In T2D, increased levels of IL-12 have also been reported as a possible contributor to endothelial dysfunction and accelerated atherosclerosis leading to the development of macrovascular complications [29]. In newly diagnosed T2D, Mishra et al. found that those with cardiovascular complications had higher levels of IL-12, independent of other serum markers [30], complementing our current findings. Interestingly, IL-12/IL-23p40s association to neurocardiac function was largely unaffected by demographic, diabetes, and cardiovascular risk factors, e.g., cholesterolemia, suggesting a robust link between the two. More studies are warranted on the association and possible causative role of IL-12/IL-23p40 in diabetic CVD. However, if our data are confirmed, IL-12/IL-23 may provide a valid cardiovascular health biomarker, constituting a potential drug target for CVD prevention.

Like neural cells, vascular endothelial cells are sensitive to hyperglycemia as glucose uptake is independent of insulin, increasing the risk of cytotoxicity. Intracellular glucose accumulation activates a cascade of metabolic pathways, promoting an inflammatory response and endothelial dysfunction [31]. Endothelial dysfunction is a critical consequence of vascular inflammation, characterised, at least partly, by elevated levels of adhesion molecules, including ICAM and VCAM. Especially the serum levels of ICAM-1 are known to be elevated in T2D and have previously shown associations with anthropometric characteristics [32]. Jude et al. investigated a small, mixed cohort of people with diabetes. They found that elevated concentrations of ICAM-1 were associated with a 4–10% increased risk of developing macrovascular disease 5 years later, which was not the case for other adhesion molecules [15]. Herder et al. took it one step further and looked at the association between ICAM-1 and heart rate variability measures in T2D [14]. They initially showed inverse associations between ICAM-1 and SDANN, rMSSD, VLF, or LF, suggesting that elevated ICAM-1 levels were associated with both lowered parasympathetic and sympathetic activity. However, only the association with SDANN persisted after multiple adjustments. Despite our slightly smaller and older cohort, our data support these findings, suggesting ICAM-1 contributes to altered neurocardiac dysfunction.

A complementary system to cardiovascular health is the lymphatic system. This all-reaching network collects excess fluid from microvascular vessels and transports it back into the circulatory system. During inflammation, the vascular endothelium can become leaky, leading to increased stress on the lymphatic system and a need for lymphangiogenesis mediated by VEGF-C [33]. However, the marker is unspecific, as recent investigation of VEGF-C has shown it to be involved in reverse cholesterol transporters in atherosclerosis and remodeling of lymphatic tissue after myocardial infects [33]. In the current study, we found increased VEGF-C levels associated with decreased response to standing ratio, a measure of adaptive mechanism to changes in blood pressure as a proxy for sympathetic integrity. This association is of particular interest as it persisted after adjusting for risk factors such as hypertension and smoking. Thus, the ability of VEGF-C to control and maintenance of tissue fluid balance, especially around the heart and blood vessels, could explain the association with adaptive blood pressure changes found in this study. Interestingly, this was the only association found in relation to cardiovascular autonomic reflex test and cardiac vagal tone. Hence, these results suggest that the parasympathetic innervation to the heart and cardiovascular autonomic neuropathy are not linked to the inflammatory profile. This is despite a low median cardiac vagal tone of 3.2 and 40% of the cohort exhibiting some level of cardiovascular autonomic neuropathy.

We explored possible influencing factors and covariates of the associations between serum markers and heart rate variability measures and found none of the covariants substantially affected the results. Despite 26% of the cohort reporting use of insulin together with other antidiabetic medications, this did not appear to have a confounding effect on the associations, suggesting that the proposed serum markers found in the current study could be investigated independently of insulin use. However, though the participant included in the cohort were diagnosed with T2D by their endocrinologist, we did not confirm their T2D status, by e.g., GAD antibody test. Similarly, glycemic control in the form of hemoglobin A1c did not affect the results, essentially affecting the results despite three in four participants being dysregulated.

This study is the first to investigate the associations between an extensive collection of inflammatory and inflammation-related serum markers and neurocardiac function in a diverse population of T2D. Although we believe our study is generally sound, there are some limitations. Firstly, the cross-sectional nature of the included cohorts prevents causational conclusions about the results. However, the strong associations between IL-12/IL-23p40 and cardiac-derived parameters warrant further exploration. Secondly, the specificity of the IL-12/IL-23p40 results is limited by the assays’ simultaneous measurement of both cytokines. Thus, though they possess similar functions, this study cannot elucidate each cytokine’s specific level and function. Thirdly, multiple factors are known to influence both inflammation level and cardiac health. For instance, it has been shown that anticoagulative medications decrease the risk of thromboembolism in diabetes [34], the triglyceride-glucose index is associated with the risk of stroke [35], and adiponectin is associated with major adverse cardiovascular events [36]. Neither of these was measured in this study, we have, however, previously shown an association between adiponectin with heart rate variability measures in type 1 diabetes [37]. Fourthly, a large proportion of the cohort used antihypertensive medication, including β-blockers, which may affect the study results. However, as hypertension is a common comorbidity of T2D, excluding these participants would decrease the generalizability of the study. Fifthly, we only investigated measures of neurocardiac function, and thus our results cannot conclude on the vascular aspect of the CVD complex. Therefore, measurements of vascular function, including those of atherosclerosis or arterial stiffness, would be beneficial to illuminate this aspect. Lastly, due to the extensiveness of the dataset, multiple testing was unavoidable and may have resulted in an inflation of the type 1 error rate. To regulate this, we lowered the inferred p-value adopting a more conservative approach, believing our explorative results could provide a basis for further research into the pathogenesis of cardiovascular function in T2D.

In conclusion, we found few but strong associations between neurocardiac function and serum markers, especially IL-12/IL-23p40, suggesting systemic changes in proinflammatory, endothelial, and lymphatic function, impacting overall cardiovascular function. Despite the study's cross-sectional design, the strong results, unaffected by covariates, propose three relatively uninvestigated markers for further exploration as cardiovascular function biomarkers in T2D. A further understanding of their underlying cardiovascular mechanisms could potentially lead to pharmacological interventions, possibly haltering or preventing neurocardiac dysfunction in the future.

## Supplementary Information

**Additional file 1: Table S1a.** Cardiovascular parameters. **Table S1b.** Neuroinflammatory parameters. **Table S2.** Associations between neuroinflammatory markers and neurocardiac function.

## Data Availability

The dataset supporting the conclusions of this article is available from the corresponding author upon reasonable request.
