# Supplementary material for: Elevated levels of interleukin-12/23p40 may serve as a potential indicator of dysfunctional heart rate variability in type 2 diabetes
Source: Cardiovasc Diabetol. 2022 Jan 6;21:5. doi: 10.1186/s12933-021-01437-w (PMC8739720; doi:10.1186/s12933-021-01437-w)
Supplement: Supplementary file 1 — Additional file 1: Table S1a. Cardiovascular parameters. Table S1b. Neuroinflammatory parameters. Table S2. Associations between neuroinflammatory markers and neurocardiac function. [file 12933_2021_1437_MOESM1_ESM.docx]

**Additional file**

**Missing data**

We could not determine all cytokines for two participants; thus they were not included in the statistical analysis. Additionally, Flt-1, PlGF, Tie-2, VEGF-D, Eotaxin, MCP-1, IL-10, IL-12/IL-23p40, and sICAM-1 were removed in one of the participants, bFGF, MDC, IL-6, IL-7, IL-8, IL-15, IL-16, IFN-γ, TNF-α, sVCAM-1, and SAA from two participants, VEGF-C, MIP-1β, TARC, VEGF, and CRP from 3 participants and IP-10 from four participants as per criteria described in the method section. These participants were included in the analysis for their remaining inflammatory markers.

A total of nine participants did not have data on heart rate variability due to a recording length below 24 hours, while 10 had a percentage of valid sinus rhythm below 90%. These participants were not included in the statistical analysis. As for the other measures of neurocardiac function, 13 participants were missing cardiac vagal tone, 2 were missing cardiac autonomic neuropathy scores, with missing data for the individual test being 3 for resting to standing, 8 for the Valsalva maneuver (some participants found it difficult to uphold the pressure needed for 15 seconds to complete this test) and 1 for expiration inhalation ratio (one participant lacked all test).

**TABLE S1a** – Cardiovascular parameters

| **Cardiovascular parameters** |  |
| --- | --- |
| Hypertension (yes) | 41% |
| Systolic BP (mmHg) | 137 (128;146) |
| Diastolic BP (mmHg) | 77 ± 9 |
| Mean arterial blood pressure | 97 ± 10 |
| Heart rate (beats/min) | 70 ± 10 |
| Cardiovascular autonomic neuropathy |  |
| Borderline (yes) | 31% |
| Established (yes) | 9% |
| Resting to standing (ratio) | 1.1 (1.1;1.2) |
| Deep breathing (ratio) | 1.2 (1.1;1.3) |
| Valsalva manoeuvre (ratio) | 1.3 (1.2;1.5) |
| Cardiac vagal tone (LVS) | 3.2 (2.1;4.6) |
| COMPASS31 | 9.8 (4.5-23.4) |
| Orthostatic intolerance (abnormal) | 32% |
| Vasomotor (abnormal) | 11% |
| Heart rate variability |  |
| SDNN | 118 ± 34 |
| SDANN | 102 ± 31 |
| SDNNi | 41 (35;54) |
| rMSSD | 27 (23;35) |
| VLF | 925 (612;1507) |
| LF | 401 (254;741) |
| HF | 210 (134;317) |
| LF/HF ratio | 0.3 ± 0.2 |
| Data is noted as mean ± standard deviation, median (25^th^-75^th^ quartile), and percentage. COMPASS: Composite autonomic symptoms score, SDNN: standard deviation of all normal interbeat intervals; SDANN: standard deviation of the averages of interbeat intervals; SDNNi: mean standard deviation of the averages of inter-beat interval for each 5-minute interval; rMSSD: root mean square of the successive differences; VLF: very low frequency; LF: low frequency; HF: high frequency; LF/HF: low/high-frequency ratio. | |

**TABLE S1b** – Neuroinflammatory parameters

| **Neuroinflammatory markers** | |
| --- | --- |
| Angiogenesis panel |  |
| bFGF (pg/mL) | 10.2 (6.4-14.9) |
| Flt-1 (pg/mL) | 124 ± 29 |
| PIGF (pg/mL) | 11.0 ± 2.3 |
| Tie-2 (pg/mL) | 4634 ± 963 |
| VEGF-C (pg/mL) | 524 ± 159 |
| VEGF-D (pg/mL) | 1272 ± 370 |
| Chemokine panel |  |
| Eotaxin (pg/mL) | 340 (260-434) |
| IP-10 (pg/mL) | 576 (444-725) |
| MCP-1 (pg/mL) | 323 ± 105 |
| MDC (pg/mL) | 1060 (888-1279) |
| MIP-β (pg/mL) | 162 ± 48 |
| TARC (pg/mL) | 275 (169-412) |
| Cytokine panel |  |
| IL-7 (pg/mL) | 8.2 (5.9-10.2) |
| IL-12 (pg/mL) | 108 (83-146) |
| IL-15 (pg/mL) | 2.6 (2.3-3.0) |
| IL-16 (pg/mL) | 208 ± 48 |
| VEGF (pg/mL) | 100 (67-150) |
| Proinflammatory panel |  |
| IFN-γ (pg/mL) | 5.2 (3.1-7.7) |
| IL-10 (pg/mL) | 0.28 (0.21-0.37) |
| IL-6 (pg/mL) | 0.92 (0.61-1.30) |
| IL-8 (pg/mL) | 12.4 (8.8-16.7) |
| TNF-α (pg/mL) | 1.5 (1.3-1.8) |
| Vascular injury panel |  |
| CRP (ng/mL) | 2769 (1038-5237) |
| sICAM-1 (ng/mL) | 486 (426-574) |
| sVCAM-1 (ng/mL) | 626 (555-743) |
| SSA (ng/mL) | 2887 (1822-6053) |
| Data is noted as mean ± standard deviation, median (25^th^-75^th^ quartile), and percentage. bFGF: basic fibroblast growth factor; Flt: fms related receptor tyrosine kinase; PIGF: phosphatidylinositol-glycan biosynthesis class F protein; Tie: tyrosine-protein kinase; VEGF: vascular endothelial growth factor; IP-10: interferon-gamma-induced protein; MDC: macrophage-derived chemokine; MIP: macrophage inflammatory protein; TARC: thymus and activation regulated chemokine; IL: interleukin; IFN: interferon; TNF: tumour necrosis factor; CRP: C-reactive protein; sICAM: soluble intercellular adhesion molecule; sVCAM: soluble vascular adhesion molecule 1; SAA: serum amyloid A. | |

**TABLE S2** - Associations between neuroinflammatory markers and neurocardiac function

|  | CVT | | CAN | | RS | | EI | | VM | |
| --- | --- | --- | --- | --- | --- | --- | --- | --- | --- | --- |
|  | R^2^ | p | R^2^ | p | R^2^ | p | R^2^ | p | R^2^ | p |
| **Angiogenesis** |  |  |  |  |  |  |  |  |  |  |
| bFGF | 0.003 | 0.65 | 0.003 | 0.51 | 0.016 | 0.22 | 0.018 | 0.20 | 0.002 | 0.65 |
| Flt-1 | 0.010 | 0.37 | 0.001 | 0.73 | 0.005 | 0.48 | 0.003 | 0.58 | 0.005 | 0.50 |
| PIGF | 0.006 | 0.47 | 0.007 | 0.34 | 0.028 | 0.11 | 0.009 | 0.35 | 0.017 | 0.22 |
| Tie-2 | 0.001 | 0.80 | 0.007 | 0.34 | 0.013 | 0.27 | 0.003 | 0.57 | 0.001 | 0.75 |
| VEGF-C | 0.001 | 0.85 | 0.001 | 0.80 | **0.083** | **0.005** | 0.000 | 0.98 | 0.001 | 0.80 |
| VEGF-D | 0.000 | 0.97 | 0.010 | 0.27 | 0.007 | 0.42 | 0.013 | 0.27 | 0.005 | 0.52 |
| **Chemokine** |  |  |  |  |  |  |  |  |  |  |
| Eotaxin | 0.003 | 0.64 | 0.000 | 0.91 | 0.006 | 0.44 | 0.009 | 0.35 | 0.000 | 0.84 |
| IP-10 | 0.023 | 0.17 | 0.000 | 0.92 | 0.007 | 0.44 | 0.023 | 0.15 | 0.014 | 0.28 |
| MCP-1 | 0.000 | 0.91 | 0.016 | 0.15 | 0.000 | 0.94 | 0.009 | 0.35 | 0.003 | 0.62 |
| MDC | 0.025 | 0.15 | 0.000 | 0.88 | 0.057 | 0.02 | 0.000 | 0.85 | 0.004 | 0.56 |
| MIP-β | 0.000 | 0.86 | 0.004 | 0.47 | 0.017 | 0.21 | 0.000 | 1.00 | 0.003 | 0.65 |
| TARC | 0.013 | 0.30 | 0.013 | 0.22 | 0.023 | 0.15 | 0.025 | 0.13 | 0.010 | 0.36 |
| **Cytokine** |  |  |  |  |  |  |  |  |  |  |
| IL7 | 0.022 | 0.18 | 0.015 | 0.20 | 0.000 | 0.93 | 0.001 | 0.80 | 0.058 | 0.02 |
| IL12IL23p40 | 0.013 | 0.15 | 0.024 | 0.09 | 0.049 | 0.03 | 0.019 | 0.19 | 0.058 | 0.02 |
| IL15 | 0.013 | 0.30 | 0.002 | 0.63 | 0.003 | 0.61 | 0.004 | 0.56 | 0.012 | 0.31 |
| IL16 | 0.002 | 0.72 | 0.004 | 0.49 | 0.001 | 0.77 | 0.002 | 0.67 | 0.003 | 0.63 |
| VEGF-A | 0.004 | 0.55 | 0.011 | 0.24 | 0.004 | 0.57 | 0.034 | 0.07 | 0.014 | 0.27 |
| **Proinflammatory** |  |  |  |  |  |  |  |  |  |  |
| IL-6 | 0.016 | 0.26 | 0.000 | 0.88 | 0.004 | 0.60 | 0.005 | 0.48 | 0.008 | 0.41 |
| IL-8 | 0.015 | 0.27 | 0.001 | 0.80 | 0.001 | 0.72 | 0.004 | 0.54 | 0.014 | 0.26 |
| IL-10 | 0.048 | 0.04 | 0.021 | 0.11 | 0.004 | 0.55 | 0.057 | 0.019 | 0.000 | 0.91 |
| IFN-γ | 0.027 | 0.14 | 0.005 | 0.44 | 0.020 | 0.18 | 0.000 | 0.85 | 0.002 | 0.69 |
| TNF-α | 0.012 | 0.32 | 0.008 | 0.31 | 0.015 | 0.25 | 0.000 | 0.85 | 0.011 | 0.33 |
| **Vascular injury** |  |  |  |  |  |  |  |  |  |  |
| CRP | 0.033 | 0.10 | 0.001 | 0.72 | 0.000 | 0.91 | 0.000 | 0.92 | 0.008 | 0.40 |
| sICAM1 | 0.004 | 0.56 | 0.000 | 0.87 | 0.035 | 0.07 | 0.001 | 0.72 | 0.001 | 0.76 |
| sVCAM1 | 0.032 | 0.10 | 0.021 | 0.11 | 0.011 | 0.31 | 0.020 | 0.17 | 0.002 | 0.70 |
| SAA | 0.011 | 0.34 | 0.000 | 0.95 | 0.009 | 0.38 | 0.001 | 0.82 | 0.001 | 0.81 |
| Data is noted as R-squared and p-values for each regression analysis. A p-value <=0.01 is considered significant and marked in bold.  CVT: cardiac vagal tone; CAN: cardiovascular autonomic reflex tests, RS: resting to standing ratio, EI: expiration/inspiration ratio; VM: Valsalva maneuver; bFGF: basic fibroblast growth factor; Flt: fms related receptor tyrosine kinase; PIGF: phosphatidylinositol-glycan biosynthesis class F protein; Tie: tyrosine-protein kinase; VEGF: vascular endothelial growth factor; IP-10: interferon-gamma-​induced protein; MCP: monocyte chemoattractant protein; MDC: macrophage-derived chemokine; MIP: macrophage inflammatory protein; TARC: thymus and activation regulated chemokine; IL: interleukin; IFN: interferon; TNF: tumor necrosis factor; CRP: C-reactive protein; sICAM: soluble intercellular adhesion molecule; sVCAM: soluble vascular adhesion molecule 1; SAA: serum amyloid A. | | | | | | | | | | |
